# Reaction of Sulfur, Hydrogen Sulfide, and Accelerators With Propylene and Butadiene^1^

**DOI:** 10.6028/jres.065A.009

**Published:** 1961-02-01

**Authors:** Frederic J. Linnig, Edwin J. Parks, Leo A. Wall

## Abstract

As part of a study of vulcanization, propylene as a model compound for natural rubber has been reacted with sulfur alone, with hydrogen sulfide alone, and with each of these materials in the presence of certain accelerators. Butadiene as a model compound for intermediate conjugated systems found in vulcanized rubber by means of infrared studies has been similarly studied. Results of mass spectrometer analyses of the volatile portions of the reaction products indicate the formation of sulfides, disulfides, and carbon-to-carbon bonds. Zinc dimethyl dithiocarbamate (ZnDMDC), a vulcanization accelerator, facilitates the formation of hydrogen sulfide from the olefin or diolefin in the presence of sulfur, and in turn promotes the reaction of hydrogen sulfide with the olefin and diolefin. The ZnDMDC-accelerated reaction of hydrogen sulfide and sulfur with the diolefin may account for the reduced conjugation observed in vulcanizates accelerated with ZnDMDC. Studies with free radical accelerators show that a mechanism other than a free radical chain mechanism is involved in the formation of diisopropyl sulfide in the reaction of propylene with sulfur (or hydrogen sulfide) and certain substances that facilitate the reactions. The same conclusion applies to the formation of a nonvolatile residue in the ZnDMDC-accelerated reaction between propylene and sulfur. Other phases of the reactions involve the formation of compounds from what appear to be free radical fragments of the original molecule. In most of the reactions, appreciable portions of the reaction products are nonvolatile.

## 1. Introduction

The processes involved in the vulcanization of natural rubber may be explored from essentially three different standpoints. From changes in the physical properties during or after vulcanization one may deduce that certain changes have occurred in the forces holding the long chain molecules together. Chemical studies, which are necessary to determine the exact nature of these forces or bonds, when applied to the polymer itself, can yield only limited information because of the difficulty of establishing the changes in chemical structure that have taken place. The use of model compounds yields much more detailed data, but it is recognized that these data may not be wholly applicable to the large polymer molecules.

Though much work of all three types has been done in the 121 years since vulcanization was discovered, no general theory of the mechanism of the reaction has as yet been agreed upon. Additional and even contradictory data are continually forthcoming. Since hydrogenated rubber will not vulcanize [1],^2^ it is apparent that the presence of the double bond in rubber must account for its reactivity in the vulcanization process. In the present work propylene was chosen as a model compound for the original olefin present in natural rubber. Its size as one of the smallest compounds that could be chosen for this purpose makes possible convenient analysis of at least part of the reaction products with the mass spectrometer.

Infrared work in which natural rubber and squalene were reacted with sulfur has indicated the likely occurrence of double bond shifts leading to the presence of 1,4, and 1,3 diolefins [2,3]. Chemical studies with maleic anhydride using infrared analysis verified the presence of the conjugated dienes [2]. Accelerators such as zinc dibutyl dithiocarbamate and tetramethylthiuram disulfide when added to rubber sulfur compounds were found to reduce the amount of conjugated double bonds [2]. More recently ZnDMDC (zinc dimethyl dithiocarbamate) has been found to have a similar effect [3]. Bateman, Glazebrook, and Moore [4] in their work with dihydromyrcene have isolated a conjugated triene, *allo*- ocimene. It will be noted that in this case the conjugation is produced not by double bond shifts but by the introduction of a new double bond. In the present work butadiene was chosen as a model compound to determine how the conjugated system, evidently present during vulcanization, would behave as an intermediate. Since hydrogen sulfide has long been considered as an intermediate in the process, its reaction with both olefin and diolefin has also been studied.

The aim of the present work was to identify the volatile products of these reactions and to provide background information for further detailed study of the individual reactions. ZnDMDC has been used as a vulcanization accelerator and di-*tert*-butyl peroxide and gamma rays have been used as free radical initiators to explore in a general way the mechanisms of the reactions.

## 2. Materials

Propylene: C. P. Grade, quoted 99.0 percent minimum purity. Mass spectrometer analysis showed about 1.0 to 1.5 percent propane.
Butadiene: Quoted 99.4 percent minimum purity.
Hydrogen sulfide: Purified grade, quoted 99.5 percent minimum purity verified by mass spectrometer analysis.
Sulfur: Purified by the method of Murphy, Clabaugh, and Gilchrist [5].
ZnDMDC (zinc dimethyl dithiocarbamate): Vulcanization accelerator.
Di-*tert*-butyl peroxide.

## 3. Experimental Procedure

Air and water were removed in the usual manner from the gaseous reagents. Specified amounts of the gaseous materials were introduced into evacuated 3-mm glass tubes 7 in. long into which the required amount of liquid or solid reagent had previously been weighed. Except in the case of the reactant blanks in table 2, about 6×10^−4^ mole of total gas was used per sample in a volume of about 0.44 ml. The quantities of reactants given in the tables are approximate.

After reaction at 130° C for 4 days, each tube was placed in a glass apparatus equipped with a glass plunger for breaking the 3-mm tubing. The glass apparatus was then evacuated and sealed. The part containing the tube was frozen in liquid nitrogen, the tube broken with the plunger, and the gases allowed to expand into the larger volume to a pressure of about 1/4 atm.

The volatile portions of the samples were analyzed in a mass spectrometer. Fractions volatile at liquid nitrogen temperature (−196° C), dry ice temperature (−78° C), and room temperature (25° C) were analyzed separately, pumping off the gases volatile at the lower temperature before distilling off the material volatile at the next higher temperature.

The nonvolatile residue of a sample in which propylene and sulfur were reacted and one in which propylene, sulfur, and ZnDMDC were reacted, were extracted with *n*-hexane in an attempt to separate the sulfurated reaction product from the remainder of the residue. A few exploratory experiments have been made on the second above-mentioned residue using infrared and gas chromatographic techniques. In other cases a change in the appearance of the original solid or the formation of oily or viscous material has been noted as the formation of a nonvolatile residue.

## 4. Results

Yields of reaction products are presented in tables 1 to 5. Except where more than one isomer of propyl sulfide is indicated, the values in the tables include small proportions of the other isomers of this compound.

It will be noted that the recovery is quite low even in cases where little nonvolatile residue is formed. The reason for this apparent low recovery is not yet clear, but it does not interfere with a qualitative and roughly quantitative interpretation of the data. In spite of the extended reaction period involved, the extent of reaction is sometimes small, a fact probably due to the gas-solid or gas-liquid-solid systems involved.

### 4.1. Propylene, Sulfur, and Accelerators

The results obtained with propylene, sulfur, and accelerators are given in table 1.

The formation of propadiene in the presence of sulfur involves loss of hydrogen—though no free hydrogen was detected—with the introduction of a new double bond. The fact that propadiene is not formed in the presence of ZnDMDC is similar to the reduction of conjugation in accelerated rubber vulcanizates [3] assuming, of course, that this conjugation is also produced by the introduction of a new double bond [4]. Diisopropyl sulfide is one of the principal volatile products formed in all four reactions and the quantity formed is not increased in the presence of the free radical accelerator, di-*tert*-butyl peroxide; it is, in fact, notably reduced in amount in the presence of gamma rays, indicating that this product of the reactions may result from something other than a free radical chain mechanism. It might be noted, however, that the work of Moore [6] on the hydrogenation of propylene suggests that isopropyl sulfide radical, if formed, could add to the central carbon atom of propylene and hence produce diisopropyl sulfide. It should be noted that the formation of the 2,5-dimethylthiophene involves the production of a carbon-to-carbon bond.

The reactant “blanks” in table 2 show that carbon disulfide is not found when ZnDMDC is heated alone or with sulfur, but is found in the presence of propylene. Table 2 indicates that hydrogen sulfide is not detected when ZnDMDC is heated alone but is detected in the presence of sulfur. This last quantity is, however, only about 0.75 percent of the amount obtained in the presence of propylene and sulfur reported in table 1.

Many workers have reported on or discussed the presence or absence of hydrogen sulfide in connection with vulcanization studies using rubber or model compounds under a variety of conditions [7–43]. Wolesensky [44] reviews some of the earlier work in this regard. Wolesensky [44] and Cummings [45] noted the evolution of hydrogen sulfide from vulcanizates between room temperature and the temperature of complete thermal decomposition. Bloomfield [46] and Farmer and Shipley [34, 36] discuss the formation of hydrogen sulfide when polysulfides are heated at different temperatures, polysulfides having been reported among the products formed in the reaction of olefins with sulfur [34, 36].

Studebaker and Nabors [7] found that purified sulfur produced appreciably less hydrogen sulfide when heated with carbon blacks than did ordinary reagent grade sulfur, and that purified sulfur heated with squalene produced only traces of hydrogen sulfide. The results of Studebaker and Nabors [7] also indicate that some hydrogen sulfide is produced when reagent grade sulfur is heated alone and that this quantity is greater than when purified sulfur is used.

Stevens and Stevens [29] reacted large quantities of sulfur (200 and 1000 phr)^3^ at 100 °C for 168 Inin a liquid mixture of *o*-and *p*-dichlorobenzenes with 20 phr zinc oxide and 20 phr zinc diethyl dithiocarbamate to produce an abundance of hydrogen sulfide, especially in the reaction employing 1000 phr sulfur, presumably due to the large excess of sulfur and the completeness of the reaction with the double bond in rubber. In our work we also used an excess of sulfur as well as long reaction periods, and in the presence of ZnDMDC recovered very little propylene.

The quantity of propane reported in column 2 of the numerical data in table 1 is close to the amount present as an impurity in the original propylene as indicated by mass spectrometric analysis.

As indicated above, ZnDMDC markedly reduces the quantity of residual propylene, thus increasing the amount of nonvolatile residue, whereas a heavy dose of gamma rays does not affect greatly the extent of overall reaction. However, there appears to be some reduction in the amount of residual propylene in the presence of the perioxide used in small amounts. Nevertheless, in the overall view, there is little evidence of a free radical chain mechanism being involved in the formation of the nonvolatile residue in the tube containing ZnDMDC. Infrared spectra of this nonvolatile residue indicate that it is not merely polypropylene. Gas chromatographic separation of the portion of this residue extractable with *n*-hexane yields at least three very broad bands and the pyrolyzate of this same material yields about 12 peaks, both results indicating a material of high molecular weight and perhaps a high degree of complexity.

The presence of methyl-*n*-hexyl sulfide, methyl isopropyl sulfide, and dimethyl trisulfide in the reaction in which the peroxide was used, suggests that free radicals do aid in the formation of sulfur compounds through fragmentation and rearrangement. However, some of the fragments may have come from the peroxide itself. The formation of methane and hydrogen in the presence of both free radical accelerators and the formation of 3-methyl- pentane, 3-methyl-1-pentene, and trans-2-pentene in the presence of gamma rays are evidences of reactions not involving sulfur.

### 4.2. Propylene, Hydrogen Sulfide, and Accelerators

The results obtained by reacting propylene with hydrogen sulfide and accelerators are given in table 3.

Little reaction takes place in the absence of an accelerator in agreement with previous findings [47] even when reaction was attempted at 180 °C. Naylor [33] gives references in which a variety of “catalysts” were employed for this type of reaction. The presence of a small amount of sulfur produces more diisopropyl sulfide than was produced in the reaction between propylene and sulfur alone (though the sulfur was present in larger amounts than here), indicating the effect of this element in promoting the reaction between propylene and hydrogen sulfide. Some isopropyl mercaptan may also be formed (see footnote c of table 3). These results are in agreement with those of Jones and Reid [47] obtained at 180 °C except that they do not report finding methyl ethyl sulfide, ethane, and ethylene. ZnDMDC produces an appreciable quantity of diisopropyl sulfide.

The information presented in section 4.1 indicates that one function of ZnDMDC in vulcanization may be to produce hydrogen sulfide in the presence of sulfur and the olefin. The data in table 3, if applied to rubber, suggest that ZnDMDC will also promote the reaction of the double bond with any hydrogen sulfide formed during ordinary vulcanization. This last reaction coupled with the “catalytic” effect of sulfur should keep the amount of free hydrogen sulfide at a low level because it is formed and reacted in the rubber where it cannot readily escape.

Failure to find additional diisopropyl sulfide when ZnDMDC was used in the propylene sulfur reaction appears to be due to the fact that too little hydrogen sulfide was formed to affect appreciably the reaction in this type of system. Furthermore some of the reaction product of ZnDMDC, propylene, and hydrogen sulfide in the presence of sulfur may be nonvolatile and appear in the residue, as it also seems to do in column 3 of the numerical data in table 3. Many workers have suggested a variety of ways in which hydrogen sulfide could play a part in vulcanization [7–9, 11, 13–15, 18–21, 23–28, 30–41, 48–51].

The amount of carbon disulfide in column 3 of the numerical data is about equal to that formed in the propylene-ZnDMDC, and hydrogen sulfide- ZnDMDC “blanks” combined (table 2).

The formation of *n*-propyl mercaptan, di-*n*-propyl sulfide, and *n*-propyl isopropyl sulfide in the presence of gamma rays indicates a considerable amount of abnormal addition contrary to Markownikoff’s rule, including the formation of *n*-propyl mercaptan as an intermediate in the production of the sulfides. This type of reaction has for some time been considered as evidence of a free radical chain mechanism [30, 52, 53]. The formation of diisopropyl sulfide with either sulfur or ZnDMDC is an indication of a mechanism other than a free radical chain reaction. A polar mechanism was indicated by Naylor [30] for the “sulfur-catalyzed” reaction of hydrogen sulfide with an olefin.

Breakdown of the original propylene is indicated by the formation of hydrogen (if not present as a residual gas in the spectrometer) in the first column of numerical data, the formation of methyl ethyl sulfide, ethane, and ethylene in the second column, and the formation of methyl isopropyl sulfide in the third column. The methyl fragment producing the methyl isopropyl sulfide in the third column may have come from the ZnDMDC. The formation of methyl ethyl sulfide, carbon disulfide, and ethylene is evidence of breakdown in the presence of gamma rays.

Some nonvolatile residue is formed in the presence of ZnDMDC, but the low recovery of hydrogen sulfide and propylene in the presence of gamma rays suggests that a large portion of these reactants has produced a nonvolatile product.

### 4.3. Butadiene, Sulfur, and ZnDMDC

The results obtained when butadiene is reacted with sulfur alone and with sulfur and ZnDMDC are given in table 4.

The formation of thiophene is facilitated somewhat by ZnDMDC. The relatively large quantity of hydrogen sulfide formed in the presence of ZnDMDC is consistent with the results of the propylene studies In this case the carbon disulfide is probably also related to a reaction between an olefin and ZnDMDC as indicated by the propylene-ZnDMDC “blank” in table 2. The remaining compounds formed in both reactions appear to result from fragmentation of the original butadiene, rearrangement, and hydrogen migration. Again, the methyl group in methyl ethyl sulfide may result from decomposition of the ZnDMDC.

Some residual butadiene exists in the presence of sulfur alone, but none when ZnDMDC is also present. This result is consistent with the reduced conjugation present in vulcanizates accelerated with ZnDMDC [3]. However, the opportunity for polymerization of the conjugated system in vulcanizing rubber should be much less than in the case of butadiene itself and should lead to larger equilibrium concentrations of conjugated systems in both types of vulcanizates. In the present work, the polymerized butadiene along with nonvolatile sulfur compounds is assumed to be present in the residues of the samples listed in table 4. Shepard, Henne, and Midgley [54] have prepared thiophene and its homologs in 6–50 percent yield by reacting 1,3 diolefins with sulfur at about 350 °C. Byproducts of the reaction at this temperature were hydrogen sulfide, carbon disulfide, and nonvolatile materials. Böttcher and Lüttringhaus [55] have prepared a trithione C_5_H_6_S_3_ in 5 percent yield from isoprene and sulfur.

### 4.4. Butadiene, Hydrogen Sulfide, and Accelerators

The results obtained when butadiene is reacted with hydrogen sulfide alone and in the presence of ZnDMDC and di-*tert*-butyl peroxide are given in table 5.

The formation of methyl ethyl sulfide and ethyl mercaptan when the two gases are reacted alone gives evidence of some reaction in contrast with the results obtained with propylene. Their formation also shows evidence of fragmentation and hydrogen migration. The small amount of residual butadiene and the presence of the unsaturated hydrocarbon C_8_H_12_ give evidence of the tendency of butadiene to polymerize and dimerize under these conditions. The relatively large quantity of residual hydrogen sulfide indicates that the major portion of the nonvolatile residue is polymer and not sulfurated reaction product.

The presence of ZnDMDC produces as usual carbon disulfide as well as a butene. Nearly all of the butadiene and all of the hydrogen sulfide seem to have produced a nonvolatile residue. It is apparent that this nonvolatile residue is not entirely polymeric in nature and must contain nonvolatile sulfurated products. Apparently ZnDMDC also promotes the reaction of hydrogen sulfide with butadiene.

The presence of the peroxide leads to the formation of di-*tert*-butyl sulfide and *tert*-butyl alcohol probably as a result of decomposition of the di-*tert*-butyl peroxide. The unsaturated hydrocarbon C_8_H_14_ is also formed as well as mixed butenes, normal butane, and methane, presumably involving fragmentation and hydrogen migration. Nearly all the butadiene and hydrogen sulfide appear to have produced a nonvolatile residue that must necessarily contain a considerable proportion of sulfurated material. Di-*tert*-butyl peroxide, like ZnDMDC strongly promotes the reaction of the diolefin and hydrogen sulfide, suggesting that ZnDMDC acts to produce a free radical chain mechanism.

Another sample not reported in table 5 contained both sulfur and ZnDMDC as accelerators of the reaction between butadiene and hydrogen sulfide. In computing the spectrum it was possible to take out about a dozen sulfurated products in addition to the usual carbon disulfide. These included a variety of sulfides (mono, di, and tri), a mercaptan from fragments of the original butadiene, thiophenes, and substituted thiophenes. The products seem to have resulted from ring closure, fragmentation of the original butadiene, rearrangement, and hydrogen migration. In this case, considerable hydrogen sulfide seemed to have been formed. Schneider, Bock, and Häusser [56] have also obtained thiophenes by reacting butadiene with hydrogen sulfide at temperatures between 420 and 600 °C using pyrites as a catalyst.

## 5. Discussion

Extensive detailed study of the individual reactions, especially of the nonvolatile portions of the reaction products, will of course be necessary before any definite overall mechanism may be deduced, but the present study suggests certain conclusions of a general nature. The application of these conclusions to typical vulcanization reactions may be limited not only by the small size of the model compounds but also by the fact that, in order to allow ample opportunity for reaction to occur in the systems studied here, large proportions of sulfur and ZnDMDC were used, and the reactions were carried out for four days at vulcanization temperatures. Furthermore, it is not known to what extent conjugated double bonds are produced before they react as intermediates.

The compounds found, including those produced through fragmentation and rearrangement indicate the formation of at least mono and disulfides and carbon-to-carbon bonds which may be sources of cross links in vulcanized rubber. Small amounts of thiophenes appear to result from the conjugated systems acting as intermediates as well as a certain amount of polymerization. Hydrogen sulfide is probably formed during vulcanization, and its formation in the reaction between sulfur and the olefin or conjugated system is probably promoted by the vulcanization accelerator ZnDMDC. Both ZnDMDC and sulfur promote the reaction of hydrogen sulfide with the olefin. The effect of ZnDMDC on the reaction of the conjugated system with sulfur and with hydrogen sulfide may account for the reduced amount of conjugation in vulcanizates accelerated with ZnDMDC [3].

The observed effect of free radical accelerators on the formation of diisopropyl sulfide in the reaction of propylene and sulfur and propylene, sulfur, and ZnDMDC as well as the normal mode of addition found in the reaction of propylene with hydrogen sulfide in the presence of sulfur or ZnDMDC indicate that something other than a free radical chain mechanism is involved in these reactions. There is very little evidence that free radicals initiate the formation of the nonvolatile residue in the reaction of propylene with sulfur and ZnDMDC, suggesting that this phase of the reaction also involves something other than a free radical chain mechanism. However, a free radical mechanism not involving a chain reaction cannot be ruled out in these cases.

In phases of the reactions in which compounds have been formed through fragmentation and rearrangement, the presence of free radicals as intermediates is at least suggested. The reaction of butadiene with hydrogen sulfide in the presence of ZnDMDC to form a nonvolatile residue may be a chain reaction involving free radicals. Linnig and Florin [57] have observed electron spin resonance absorption in rubber-sulfur vulcanizates and in rubber heated alone in air under vulcanizing conditions, indicating the presence of free radicals.

## Figures and Tables

**Table 1:** Reaction of propylene, sulfur, and accelerators^a^

Products	Yield^b^ in presence of accelerators			
	No accelerator	ZnDMDC	Di-*tert*-butyl peroxide^c^	Gamma rays^d^
				
Diisopropyl sulfide	0.66	0.61	0.27	0.024
Diisopropyl disulfide	…………………	e.1	e.013	…………………
Mcthyl-*n*-hexyl sulfide	…………………	…………………	e.014	…………………
Methyl isopropyl sulfide	…………………	…………………	e.004	…………………
Dimethyl trisulfide	…………………	…………………	e.003	…………………
2-Methylthiophene	…………………	…………………	…………………	.0014
2,5-Dimethylthiophene	…………………	.04	.017	…………………
2,3,4-Trimethylthiophene	…………………	…………………	…………………	.0041
Carbon disulfide	…………………	2.0	…………………	…………………
Hydrogen sulfide	…………………	1.6	…………………	…………………
3-Methylpentane	…………………	…………………	…………………	.017
3-Methyl-1-pentene	…………………	…………………	…………………	.021
Trans-2-pentene	…………………	…………………	…………………	.013
Propane	…………………	e1.1	…………………	…………………
Propadiene	e.4	…………………	e.2	…………………
Methane	…………………	…………………	e.038	.00086
Hydrogen	…………………	…………………	e.0008	.018
Propylene	f48.8	e.3	34.7	45.9
Nonvolatile residue	Slight	Considerable	Yes	Yes

a The materials were heated in sealed tubes at 130 °C for 4 days. About 0.025 g of propylene was used with about 0.1 g of sulfur or sulfur plus ZnDMDC (zinc dimethyl dithiocarbamate) in a 2:1 ratio. About 0.002 g of di-*tert*-butyl peroxide was used in the experiment with this catalyst. The reactants were exposed at 130 °C to a 1700-curie cobalt-60 source in the gamma radiation experiment.

b $\text{Yield}=\frac{\text{number of moles of gaseous product or residual propylene}\times 100}{\text{initial number of moles of propylene}}$; Average values of analyses of duplicate tubes are given except where noted.

c The room temperature fractions of these tubes contained large portions of unidentified material.

d Based on one reaction tube.

e Value based on analysis of one tube; not found in analysis of second tube.

f Value based on analysis of one tube; found in second tube, but not computed quantitatively.

**Table 2:** Reaction of zinc dimethyl dithiocarbamate (ZnDMDC) with other reactants^a^

Products	Yield^b^ in presence of ZnDMDC			
	ZnDMDC alone^c^	Sulfur (0.067 g)	Propylene (0.025 g)	Hydrogen sulfide (0.014 g)
				
Methyl mercaptan	…………………	…………………	…………………	0.09
Carbon disulfide	…………………	…………………	1.7	d.51
Hydrogen sulfide	…………………	e0.012	…………………	60.7
Propane	…………………	…………………	1.5	…………………
Propylene	…………………	…………………	82.9	…………………
Nonvolatile residue	No	Yes	Possibly	Yes

a The materials were heated in sealed tubes at 130 °C for 4 days. About 0.033 g of ZnDMDC was used in each experiment.

b $\text{Yield}=\frac{\text{number of moles of gaseous product or residual gas}\times 100}{\text{initial number of moles of gaseous reactant}}$.

c Analysis showed small amounts of C_4_H_6_ in all fractions and C_8_H_12_ in the room temperature fraction, evidently as contamination in the spectrometer.

d The hydrogen sulfide used probably contained about 0.1 percent carbon disulfide as an impurity.

e Calculated relative to 0.025 g of propylene ordinarily used.

**Table 3:** Reaction of propylene, hydrogen sulfide, and accelerators^a^

Products	Yield^b^ in presence of accelerators			
	No accelerator	Sulfur (0.0023 g)	ZnDMDC (0.033 g)	Gamma rays
				
Di-*n*-propyl sulfide	…………………	…………………	…………………	2.9
*n*-Propyl isopropyl sulfide	…………………	…………………	…………………	2.4
Diisopropyl sulfide	…………………	4.6	4.9	…………………
Methyl isopropyl sulfide	…………………	…………………	0.14	…………………
Methyl ethyl sulfide	…………………	c0.72	…………………	0.15
*n*-Propyl mercaptan	…………………	…………………	…………………	1.9
Carbon disulfide	…………………	…………………	2.3	0.06
Propane	0.4	.98	…………………	.78
Ethane	…………………	.33	…………………	…………………
Ethylene	…………………	.33	…………………	.20
Hydrogen	Trace	…………………	…………………	…………………
Propylene	28.4	17.0	46.1	11.3
Hydrogen sulfide remaining mole %	44.3	24.4	38.0	10.0
Nonvolatile residue	No	Probably	Yes	Yes

a The materials were heated in sealed tubes at 130 °C for 4 days. About 0.017 g of propylene was used with about 0.007 g of hydrogen sulfide in each experiment. The reactants were exposed at 130 °C to a 1700-curie cobalt-60 source in the gamma radiation experiment.

b $\text{Yield}=\frac{\text{number of moles of gaseous product or residual propylene}\times 100}{\text{initial number of moles of propylene}}$.

c Analysis shows about 50 percent of this constituent could be isopropyl mercaptan.

**Table 4:** Reaction of butadiene, sulfur, and zinc dimethyl dithiocarbamate (ZnDMDC)^a^

Products	Yield^b^	
	Sulfur (0.1 g)	Sulfur (0.067 g) ZnDMDC (0.033 g)
		
Methyl ethyl sulfide	…………………	0.044
Thiophene	0.12	.68
Carbon disulfide	…………………	.14
Hydrogen sulfide	.036	6.17
Butene	.87	…………………
Ethylene	.14	…………………
Diacetylene	.036	…………………
Methane	…………………	.14
Butadiene	.67	…………………
Nonvolatile residue	Yes	Yes

a The materials were heated in sealed tubes at 130 °C for 4 days. About 0.032 g of butadiene was used in each experiment.

b $\text{Yield}=\frac{\text{number of moles of gaseous product or residual butadiene}\times 100}{\text{initial number of moles of butadiene}}$.

**Table 5:** Reaction of butadiene, hydrogen sulfide, and accelerators^a^

Products	Yield^b^ in presence of accelerators		
	No accelerator	ZnDMDC (0.033 g)	Di-tert-butyl peroxide^c^ (0.002 g)
			
Di-*tert*-butyl sulfide	…………………	…………………	0.06
Methyl ethyl sulfide	0.05	…………………	…………………
Ethyl mercaptan	.18	…………………	…………………
Carbon disulfide	…………………	0.66	…………………
*terf*-Butanol	…………………	…………………	2.9
C_8_H_14_	…………………	…………………	0.02
C_8_H_12_	3.3	…………………	…………………
*n*-Butane	…………………	…………………	.064
C_4_H_8_	…………………	1.03	.55
Methane	…………………	…………………	.25
Butadiene	3.9	d0.084	d.21
Hydrogen sulfide remaining…mole %	54.3	…………………	.62
Nonvolatile residue	Probably	Yes	Yes

a The materials were heated in sealed tubes at 130 °C for 4 days. About 0.022 g of butadiene, and 0.007 g hydrogen sulfide was used in each experiment.

b $\text{Yield}=\frac{\text{number of moles of gaseous product or residual butadiene}\times 100}{\text{initial number of moles of butadiene}}$.

c The room temperature fraction of this tube contained a large portion of unidentified material.

d Assumed on logical grounds to be 1,3-butadiene; mass spectrometer analysis indicates possibility of its being 1,2-butadiene.

## Footnotes

2 Figures in brackets indicate the literature references at the end of this paper.

3 phr=parts per hundred parts of rubber.
